# Germline HLA-B evolutionary divergence influences the efficacy of immune checkpoint blockade therapy in gastrointestinal cancer

**DOI:** 10.1186/s13073-021-00997-6

**Published:** 2021-11-03

**Authors:** Zhihao Lu, Huan Chen, Xi Jiao, Yujiao Wang, Lijia Wu, Huaibo Sun, Shuang Li, Jifang Gong, Jian Li, Jianling Zou, Keyan Yang, Ying Hu, Beibei Mao, Lei Zhang, Xiaotian Zhang, Zhi Peng, Ming Lu, Zhenghang Wang, Henghui Zhang, Lin Shen

**Affiliations:** 1 Department of Gastrointestinal Oncology, Key Laboratory of Carcinogenesis and Translational Research (Ministry of Education), Peking University Cancer Hospital & Institute, Fu-Cheng Road 52, Hai-Dian District, Beijing, 100142 People’s Republic of China; 2 Genecast Biotechnology Co., Ltd., 88 Danshan Road, Xidong Chuangrong Building, Suite D-401, Xishan District, Wuxi City, Jiangsu 214104 People’s Republic of China; 3 Biomedical Innovation Center, Beijing Shijitan Hospital, School of Oncology, Capital Medical University, Beijing, People’s Republic of China

**Keywords:** Immune checkpoint blockade, Gastrointestinal cancer, HLA genotype, HLA-I evolutionary divergence, Tumor mutational burden

## Abstract

**Background:**

The human leukocyte antigen class I (HLA-I) genotype has been linked with differential immune responses to infectious disease and cancer. However, the clinical relevance of germline HLA-mediated immunity in gastrointestinal (GI) cancer remains elusive.

**Methods:**

This study retrospectively analyzed the genomic profiling data from 84 metastatic GI cancer patients treated with immune checkpoint blockade (ICB) recruited from Peking University Cancer Hospital (PUCH). A publicly available dataset from the Memorial Sloan Kettering (MSK) Cancer Center (MSK GI cohort) was employed as the validation cohort. For the PUCH cohort, we performed HLA genotyping by whole exome sequencing (WES) analysis on the peripheral blood samples from all patients. Tumor tissues from 76 patients were subjected to WES analysis and immune oncology-related RNA profiling. We studied the associations of two parameters of germline HLA as heterozygosity and evolutionary divergence (HED, a quantifiable measure of HLA-I evolution) with the clinical outcomes of patients in both cohorts.

**Results:**

Our data showed that neither HLA heterozygosity nor HED at the HLA-A/HLA-C locus correlated with the overall survival (OS) in the PUCH cohort. Interestingly, in both the PUCH and MSK GI cohorts, patients with high HLA-B HED showed a better OS compared with low HLA-B HED subgroup. Of note, a combinatorial biomarker of HLA-B HED and tumor mutational burden (TMB) may better stratify potential responders. Furthermore, patients with high HLA-B HED were characterized with a decreased prevalence of multiple driver gene mutations and an immune-inflamed phenotype.

**Conclusions:**

Our results unveil how HLA-B evolutionary divergence influences the ICB response in patients with GI cancers, supporting its potential utility as a combinatorial biomarker together with TMB for patient stratification in the future.

**Supplementary Information:**

The online version contains supplementary material available at 10.1186/s13073-021-00997-6.

## Background

The emergence of immune checkpoint blockade (ICB) therapy that targets programmed cell death protein 1/programmed cell death ligand 1 (PD-1/PD-L1) or anti-cytotoxic T lymphocyte-associated antigen-4 (CTLA-4) has markedly revolutionized the therapeutic landscape of patients with metastatic cancers [1, 2]. However, the clinical effectiveness of ICB treatment is still not satisfactory, especially in advanced gastrointestinal (GI) cancers [3]. To date, most studies that aimed to predict the therapeutic response to ICB treatments have focused on the intrinsic properties of tumor cells, including tumor mutational burden (TMB) [4] and microsatellite instability (MSI)/mismatch repair (MMR) status [5] and properties reflecting the immune phenotype, such as PD-L1 expression [6] and immune cell infiltration [7]. However, how germline genetics influence the efficacy of ICB therapy in GI cancer has been much less explored.

Theoretically, the fundamental basis for the ICB response is tumor immunogenicity, which mainly depends on the antigenicity of the tumor and the efficiency of antigen presentation [8]. In particular, the cell surface presentation of tumor-derived neoantigens by human leukocyte antigen class I (HLA-I) molecules is a critical process for recognition by cytolytic T cells [9, 10]. As the most polymorphic genes in the human genome, the HLA genes have a genetically predetermined background and play an essential role in host immune response. Variants of the HLA genes can possibly shape the sequence repertoire of neopeptides presented and influence the T cell receptor (TCR) repertoire during their continuous interaction [11]. Heterozygous HLA-I genotypes have been shown to confer heterozygote advantages in infectious diseases [12–14], autoimmune diseases [15] and tumor development [16]. Most recently, the HLA-I genotype and the concomitant sequence divergence between HLA-I alleles have been linked with immunotherapy efficacy in melanoma and non-small-cell lung cancer (NSCLC) [17, 18]. These studies present evidence that an HLA-I genotype with two alleles with higher sequence divergence, known as the HLA-I evolutionary divergence (HED), may enable the presentation of more diverse immunopeptidomes and hence facilitate subsequent T cell recognition and the adaptive immune response.

To further refine both host and tumor genomic contributions to the therapeutic response to checkpoint blockade, we evaluated the DNA sequencing and clinical data from two independent GI cancer cohorts receiving ICB immunotherapy. We report here the predictive and prognostic significance of the germline HLA-I allele divergence, and its interrelationship with tumor genomic determinants and immune-related gene expression profiles.

## Methods

### Study design and cohorts

#### PUCH GI cancer cohort

We retrospectively collected and analyzed the data of 84 metastatic GI patients treated with ICB in the Department of GI Oncology, Peking University Cancer Hospital & Institute (PUCH), between August 1, 2015, and May 24, 2019. The blood samples and tumor tissues were additionally collected from the clinical trials (NCT02825940 [19], NCT02915432 [20], NCT03167853 [21], NCT03472365 [22], NCT02872116 [23], NCT03713905 [24], NCT03736889 [25], NCT03667170 [26], and CTR20160872 [27]) and were newly analyzed. All patients met the following criteria: (1) diagnosis of metastatic gastrointestinal cancer with failed standard treatment; (2) patients received at least one cycle of PD-1/PD-L1 inhibitors; and (3) patients with eligible blood samples, tumor sample, and adequate clinical information. For PUCH cohort, the cancer types included gastric cancer (GC, 33.3%), esophageal squamous cell carcinoma (ESCC, 29.8%), colorectal cancer (CRC, 22.6%), and other types, including pancreatic neuroendocrine tumors (PanNETs), gastrointestinal-NETs, and cholangiocellular carcinoma. Among all patients, 66 (78.6%) were treated with anti-PD-1 therapy, 18 (21.4%) were treated with anti-PD-L1 therapy. Patients’ characteristics are summarized in Additional file 1: Table. S1. We have obtained the Ethics approval from the medical ethics committee of Peking University Cancer Hospital (2020MS01). All patients in this study provided written informed consent for their additional samples to be used in our translational research.

**Fig. 1:**
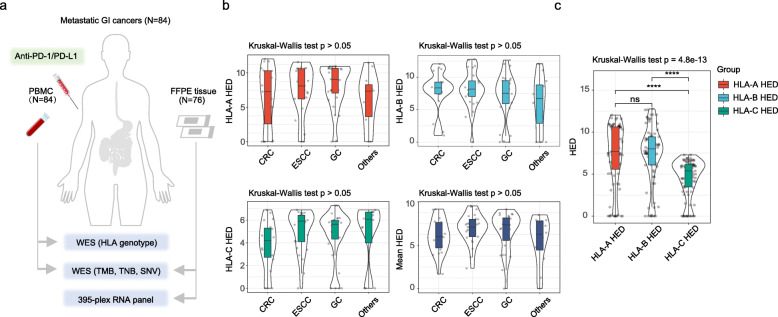
Landscape of classic HLA class I evolutionary divergence in advanced GI cancer. **a** Schematic diagram of the PUCH study design. The HLA genotype and HED were obtained from 84 patients, and FFPE samples from 76 patients were subjected to WES analysis and RNA profiling. **b** Distributions of HED at HLA-A, HLA-B, HLA-C, and mean HED across different GI cancer types in the PUCH cohort. **c** Comparison of HED distributions among HLA-A, HLA-B, and HLA-C heterozygous genotypes. ****p < 0.0001. Kruskal-Wallis test

Whole-exome sequencing (WES) analysis was performed on the peripheral blood mononuclear cell (PBMC) samples from all 84 patients. Tumor specimens were obtained from only a subset of patients from this cohort (*n* = 76), and they were subjected to WES and immune oncology-related RNA profiling (Fig. 1a) to delineate the genomic landscape and immunological phenotype. The baseline and treatment characteristics of all patients are depicted in Additional file 1: Table. S1. Tumor burden was measured by imaging studies or physical examinations according to the Response Evaluation Criteria in Solid Tumors (RECIST) v1.1 and iRECIST [28, 29]. Efficacy was defined as durable clinical benefit [DCB: complete response (CR), partial response (PR), and stable disease (SD) lasting for ≥ 24 weeks] or no durable benefit [NDB: progressive disease (PD) or SD that lasted < 24 weeks] [30]. A prognostic analysis was conducted on patients who had follow-up data for overall survival (OS) and progression-free survival (PFS).

#### MSK GI cancer cohort

To validate the prognostic value of HLA-I HED, HLA typing data, TMB values and clinical information of patients with GI cancer were obtained from a previous study from the Memorial Sloan Kettering Cancer Center (MSK GI cohort) [17]. The MSK GI cancer cohort comprised GC (4.8%), ESCC (17.9%), CRC (50.0%), pancreatic cancer (17.9%), and hepatobiliary cancer (9.5%) patients, all of whom received drugs targeting CTLA-4, PD-1/PD-L1, or a combination of both at the Memorial Sloan Kettering Cancer Center (MSKCC). Tumors were subjected to targeted next-generation sequencing (NGS) (MSK-IMPACT) [31]. Altogether, 84 GI cancer samples from the MSK cohort were included in the prognostic analysis for OS.

### HLA-I genotyping and HED calculation

For the 84 patients in the PUCH GI cancer cohort, HLA-I genotyping from normal germline DNA exome sequencing data was performed as following (Additional file 2: Supplementary methods). Briefly, reads within the HLA gene region were extracted from the Binary Alignment Map (BAM) file and then imported input into HLA-HD (v 1.2.0.1) for analysis of HLA allele type. HED was calculated according to previous investigations [18, 32, 33]. Briefly, protein sequences of each HLA class I allele (HLA-A, HLA-B and HLA-C) were obtained from the IMGT/HLA database [34]. Exons 2 and 3, which form the variable region in the peptide-binding groove, were selected following annotation with the Ensemble database [35]. Next, the HED were calculated using the Grantham distance metric implemented in Pierini and Lenz [33]. Specifically, to obtain the HED value for each HLA-I gene, a custom Perl script and two input files were queried: a FASTA file with aligned HLA alleles and a specific amino acid distance matrix [32]. The mean HED was calculated as the mean divergence at HLA-A, HLA-B and HLA-C. In all, we studied the associations of two parameters of germline HLA (heterozygosity and HED at each HLA locus) with the clinical outcomes of patients.

### TMB, copy number alteration (CNA) and neoantigen assessments

Tumor samples and paired blood cell samples from the PUCH GI cancer cohort (*n* = 76) were subjected to DNA extraction and WES analysis (Additional file 2: Supplementary methods). Somatic alterations were filtered with matched patient’s whole blood controls to remove germline mutations. Somatic SNVs were further selected with the following filters: (i) exonic regions; (ii) nonsynonymous SNV or stopgain mutation type; (iii) depth > = 40; (iv) allele frequency > = 0.03; and (v) allele frequency < = 0.002 in the Exome Aggregation Consortium (ExAC) database and Genome Aggregation Database (gnomAD) and 1000 Genomes. Next, TMB of each tumor sample was calculated by using the following formula: Absolute mutation counts * 1000000/total num of exonic base with depth larger than 40X. TMB was measured in mutations per Mb.

Based on the somatic SNVs and HLA typing results of its paired germline sample generated via OptiType [36, 37], neoantigens were predicted through software netMHCpan-4.0. For the copy number analysis, blood cell samples from patients were used as paired controls, and the CONTRA assay was used to call copy number variations from the tumor samples [38]. CNA burden was defined as the total number of genes with copy number gains or losses [39].

### MMR/MSI status testing

We performed the IHC staining on FFPE sections using monoclonal anti-mutL homolog 1 (1:60; Clone ES05, Gene Tech, Inc., South San Francisco, CA, USA), anti-mutS homolog 2(1:40; Clone 25D12, Gene Tech), anti-mutS homolog 6 (1:50; EP49, Gene Tech), and PMS1 homolog 2 (1:40; Clone EP51, Gene Tech). Tumors lacking at least one of the four proteins were defined as MMR deficiency (dMMR). In some cases, MSI status was assessed by a single multiplex PCR, containing five microsatellite loci (BAT-25, BAT-26, D2S123, D5S346, and D17S250) [40, 41]. Instability at two or more of the loci was defined as MSI-H; instability at one locus and no instability at any locus were defined as MSS [42].

### RNA immune oncology (IO) panel sequencing

For 76 GI cancer patients in the PUCH GI cancer cohort, gene expression profiling was conducted using a RNA IO panel sequencing to determine the expression of 395 immune-related genes simultaneously as previously reported [43]. For the NGS measurements to be comparable for evaluations, the background-subtracted read counts were subsequently normalized to reads per million (RPM) values as previously described [44]. Briefly, RNA was extracted, reverse transcribed, amplified, and ligated to fluorescent barcodes. The pooled libraries were sequenced on the Ion S5 530 chip (Thermo Fisher Scientific). For data analysis, 1–2 M reads per sample were obtained. Gene expression level was initially determined as RPM. For data normalization, RPM of 10 housekeeping (HK) genes from an internal control sample was applied to determine the normalization ratio using to normalize RPM counts for all genes in each test sample (Additional file 2: Supplementary methods).

### Statistical analysis

To quantify the differences in non-normally distributed quantitative variables, a Kruskal-Wallis test was applied when more than two subgroups were analyzed [45]. Survival analyses were performed using the survival and survminer R packages [46]. Germline zygosity and HED at each of the HLA-A, HLA-B and HLA-C genes; TMB; and MSI status were analyzed for associations with OS or PFS using a Kaplan-Meier survival analysis. Specifically, in the PUCH cohort, HED at each of the HLA genes or mean HED were dichotomized for OS using the optimal cutoff values determined by using the maximally selected rank statistics (‘maxstat’ method from the “surv_cutpoint” function of the “survminer” R version 3.6.1). For each HED, the patients were dichotomized into low-HED and high-HED subgroups with differential risk for OS. The DCB rates between the low-HED and high-HED subgroups were then compared using a chi-squared test. For TMB, we first analyzed its association with DCB rate, calculated an optimal cutpoint by using the Youden index, and subsequently TMB-high was designated as **>** 5.22 mut/Mb. To examine the correlation between continuous variables, we determined Spearman's rank correlation coefficient using the “ggstatsplot” or “corrplot” R package [47, 48]. Differentially expressed genes (DEGs) between HLA-B HED high and low subgroups of the RNA IO panel sequencing data (log2-transferred nRPM value) was performed by using the limma R package [49]. The results were filtered using thresholds of [log2 fold change] > 0.5849 and a *p* value < 0.05. The DEGs were then subjected to pathway enrichment analysis by using the enrichPathway function from the ReactomePA R package [50].

## Results

### Landscape of HEDs at HLA-A, HLA-B, and HLA-C in GI cancer cohort

Motivated by the divergent allele advantage of the HLA-I genotype [18, 33], we sought to comprehensively delineate the landscape of classic HLA class I genes in GI cancer and investigate their implications for immunotherapy. HLA heterozygosity and HED at each of the HLA-A, HLA-B, and HLA-C genes were assessed in the PBMC samples (*n* = 84) using the WES analysis in the PUCH GI cancer cohort (Fig. 1a). First, we observed no remarkable differences in the HED of HLA-A, HLA-B, HLA-C, or the mean-HED among different GI cancer types (Kruskal-Wallis test, Fig. 1b).

Next, when comparing the distribution patterns of HED for each HLA-A, HLA-B and HLA-C heterozygous genotype in the GI cancer cohort, we found that HLA-C pairwise divergences were significantly lower relative to the HLA-A and HLA-B pairwise divergences (Fig. 1c). This observation was in accordance with previous studies that described HLA-C as the most recently evolved gene [51, 52]. No significant difference in HED was observed between HLA-A and HLA-B alleles (Fig. 1c).

### High HLA-B HED is associated with improved DCB and OS in patients with GI cancers receiving ICB treatment

Zygosity at HLA-I genes has been linked with the clinical outcome of cancer patients treated with ICBs, especially in melanoma and NSCLC [17, 18]. We therefore explored its functional relevance to immunotherapy in the PUCH GI cancer cohort. Unexpectedly, heterozygosity at HLA-A, HLA-B, or HLA-C was not associated with improved OS or PFS when compared with homozygosity at each locus (Additional file 3: Fig. S1A and 1B).

**Fig. 2:**
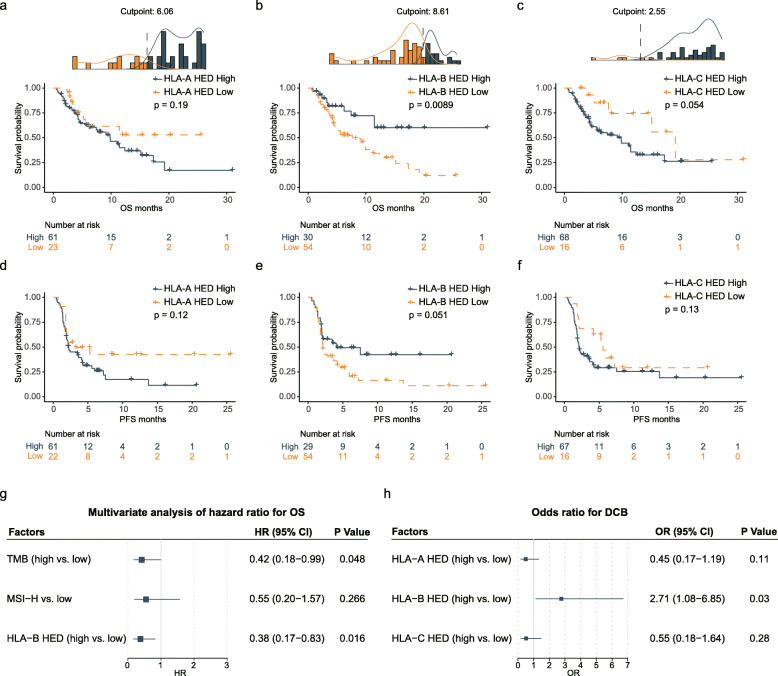
Associations between HED and immunotherapeutic efficacy and the prognosis. **a–c** Kaplan-Meier survival analysis comparing OS between patients with high and low HED at each locus: HLA-A (**a**), HLA-B (**b**), and HLA-C (**c**). Patients were dichotomized into low-HED and high-HED subgroups with differential risks for OS by using the optimal cutoff values determined by the “surv_cutpoint” function of the “survminer” R package. **d–f** Kaplan-Meier survival analysis comparing PFS between patients with high and low HED at each locus: HLA-A (**d**), HLA-B (**e**), and HLA-C (**f**) (PFS information was available for 83 patients). **g** Forest plot showing the HRs and 95% CIs for the associations of potential prognostic factors (HLA-B HED, TMB, and MSI) with OS in multivariable Cox proportional hazards model (all information was available for 76 patients). **h** Forest plot showing the ORs and 95% CIs for the associations of germline determinants with DCB

Previously, HLA divergence, especially mean HED of HLA class I genes, has been shown to impact immunotherapeutic efficacy [18]. We then examined whether the germline HED of HLA class I was associated with clinical outcomes in our cohort. First, we calculated the optimal cutoff point for OS for each HLA-I gene using the “surv_cutpoint” function from the “survminer” R package (Fig. 2). Intriguingly, patients with a high level of HLA-B HED (cutpoint: 8.61) experienced a remarkable prolonged OS compared with patients with a low level (Fig. 2b). Similarly, prolonged PFS was observed after ICB in patients with high HLA-B HED (cutpoint: 8.61, Fig. 2e). On the other hand, higher levels of and HLA-C HED (cutpoint 2.55) and HLA-A HED (cutpoint 6.06) even showed opposite trend towards poor OS (*p* = 0.054, Fig. 2c) and PFS (*p* = 0.12, Fig. 2d), respectively. Previous investigations presented evidence that mean HED is associated with the response to ICBs in melanoma and NSCLC [18]; we therefore determined the optimal cutoff point for OS for mean HED using the “surv_cutpoint” function. However, the optimal cutpoint of high mean HED only stratify 9 patients with better OS, and these patients did not show any improvements in PFS when compared with the mean-HED low subgroup (Additional file 3: Fig. S2), suggesting that HLA divergence in GI cancers may play different roles from that of melanoma or NSCLC.

Moreover, we performed univariable (Additional file 3: Fig. S3) and multivariable Cox regression analysis (Fig. 2g) on patients with available information. Our data showed that HLA-B HED is a prognostic factor for OS in the PUCH cohort (Fig. 2g), independent of TMB (Youden index: 5.22 mut/Mb) and MSI status.

Furthermore, we determined the odds ratio (OR) for DCB in different subgroups according to zygosity or the HED level at each HLA-I gene. The association between the DCB rate and heterozygosity was not significant at any HLA-I gene (Additional file 3: Fig. S1C), nor was the association between high HED at the HLA-A or HLA-C locus (Additional file 3: Fig. S4A and 4C) significant. Notably, the DCB rate was significantly higher in GI cancer patients with high HLA-B HED (cutpoint: 8.61) than in patients with low HLA-B HED (53% vs. 30%, chi-squared test, *p* < 0.05, Fig. 2 h and Additional file 3: Fig. S4B).

In addition, to test the hypothesis that HLA-B HED might be a potential germline genomic determinant for predicting the response to immunotherapy, we examined the prognostic effect of HLA-B HED in the MSK GI cohort. We observed no significant difference in the distribution pattern of HED at any HLA-I gene between the PUCH and MSK GI cancer patients (Additional file 3: Fig. S5A). Interestingly, when using the same cutpoint (cutpoint 8.61) calculated from the PUCH cohort, the patients with high HLA-B HED from the MSK GI cohort experienced a trend towards a favorable OS (Additional file 3: Fig. S5B, log-rank test *p* = 0.092). Undoubtedly, when we calculated the optimal cutoff (cutpoint 10.19) for the MSK GI cohort, the HLA-B HED high subgroup experienced a markedly improved survival after ICB treatment (Additional file 3: Fig. S5C, log-rank test *p* = 0.016). In accordance with the observations in the PUCH cohort, no significant correlation was identified between OS and the HED at the HLA-A or HLA-C locus in the MSK GI cohort (Additional file 3: Fig. S5D and 5E).

Taken together, our results indicated that the germline divergent allele advantage of the HLA-B gene, and not HLA-I zygosity, may influence the therapeutic efficacy and clinical outcomes of GI cancer patients receiving ICB therapy.

### High HLA-B HED correlated with favorable OS in both the MSS and MSI-H subpopulations

MSI-H/deficient mismatch repair (dMMR) has emerged as a potential biomarker for PD-1/PD-L1 blockade in GI cancer; we therefore performed a subgroup analysis in the PUCH cohort to test whether the prognostic value of germline HLA-B HED remains regardless of MSI status. As expected, patients with MSI-H/dMMR tumors revealed better clinical outcomes relative to those with Microstate stability (MSS) tumors (Additional file 3: Fig. S6).

**Fig. 3:**
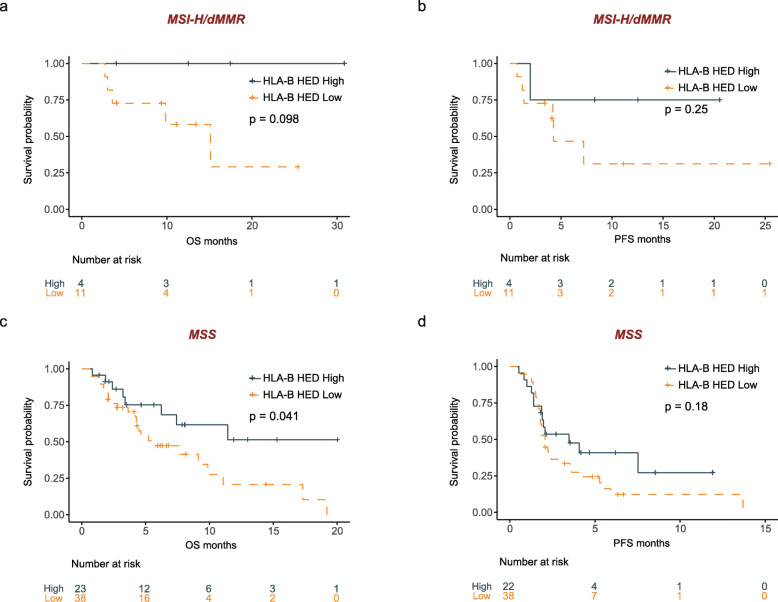
Comparison of survival distributions by HED levels in different subpopulations in the PUCH cohort. **a**, **b** Kaplan-Meier survival analysis comparing the OS (**a**) and PFS (**b**) curves between the high-HLA-B HED and low-HLA-B HED subgroups of patients with MSI-H GI cancer (*n* = 15). **c**, **d** Kaplan-Meier survival analysis comparing the OS (**c**) and PFS (**d**) curves between the high-HLA-B HED and low-HLA-B HED subgroups of patients with MSS GI cancer (*n* = 61). For the MSS subgroup, only 60 patients had PFS information (the subgroup survival analysis was not performed in the MSK GI cohort since the MSI status was not available when downloaded)

Next, we examined the associations between the HLA-B HED levels and clinical outcomes in the MSI-H/dMMR and MSS subpopulations (Fig. 3). Among the 15 patients with MSI-H/dMMR tumors, those with high HLA-B HED tended to experience prolonged OS (Fig. 3a, log-rank test *p* = 0.098) and PFS (Fig. 3b, log-rank test *p* = 0.25), although the difference did not reach statistical significance. We speculate that a larger sample size may help verify the prognostic significance of HLA-B HED.

We also examined the impact of HLA-B HED on the clinical outcomes of MSS GI cancer patients. Notably, the patients with high HLA-B HED showed a pronounced trend towards prolonged OS (Fig. 3c, log-rank test *p* = 0.041) and PFS (Fig. 3d, log-rank test *p* = 0.18).

In summary, HLA-B HED may function as a potential germline genomic determinant for predicting clinical outcomes in GI cancer patients with either MSI-H or MSS tumors after ICB treatment.

### Joint utility of germline HLA-B HED and TMB for patient stratification

**Fig. 4:**
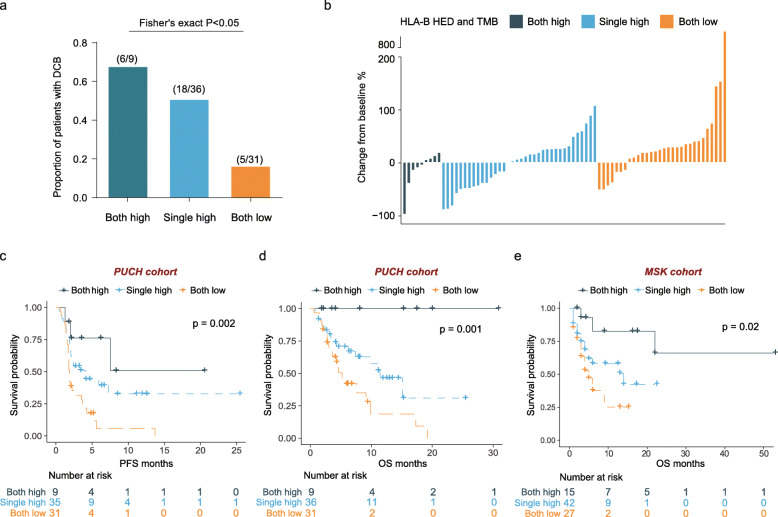
Joint utility of HLA-B HED and TMB in predicting immunotherapeutic outcomes of GI cancer patients. **a** Proportions of patients with DCB calculated within each of the three indicated subgroups. **b** Waterfall plot of tumor response to ICB according to the joint biomarker (HLA-B HED and TMB). The *Y*-axis represents the percentage of maximum tumor change from baseline according to RECIST 1.1. **c** Kaplan-Meier survival analysis of PFS among patients within each of the three indicated subgroups in the PUCH cohort (*n* = 75, the PFS information was not available for one patient). **d** Kaplan-Meier survival analysis of OS among patients within each of the three indicated subgroups in the PUCH cohort (*n* = 76). **e** Kaplan-Meier survival analysis of OS among patients within each of the three indicated subgroups in the MSK GI cohort (*n* = 84, the MSK GI cohort included 84 patients in all). For both cohorts: Both high, HLA-B HED > 8.61 and TMB > 5.22 mut/Mb; single high, HLA-B HED > 8.61 or TMB > 5.22 mut/Mb; and both low, HLA-B HED ≤ 8.61 and TMB ≤ 5.22 mut/Mb

The effect of both high mean HED (calculated as the mean divergence at HLA-A, HLA-B, and HLA-C) and high TMB on OS after ICB has previously been reported to be more pronounced than the effect of either alone in melanoma [18]. We thus sought to explore the combinatorial utility of HLA-B HED and TMB in the GI cancer cohort. We determined the optimal cutoff point for TMB in the PUCH cohort by using the Youden index in receiver operating characteristic (ROC) analysis for DCB (Additional file 3: Fig. S7A-B). As expected, in the PUCH cohort, TMB-high (> 5.22 mut/Mb) patients experienced longer median OS and PFS time than TMB-low patients (Additional file 3: Fig. S7C and 7D). When 76 patients were classified into three subgroups based on TMB and HLA-B HED (both high, single high and both low), the proportion of DCB patients was remarkably higher in the TMB-high/HLA-B HED-high (both high) subgroup (6 of 9) than in the subgroup where both were low (5 of 31) (Fig. 4a, Fisher’s exact test *p* < 0.05). This effect was consistent with the percentages of maximal tumor reduction in all three subgroups of patients after ICB treatment (Fig. 4b). Moreover, the median OS and PFS times of the TMB-high/HLA-B HED-high (both high) subgroup was significantly longer than those of the other two subgroups (Fig. 4c and 4d, log-rank test *p* < 0.01 for all comparisons). Strikingly, when using the same cutoff values as for the PUCH cohort, a similar finding was also observed in the MSK GI cancer cohort, in which TMB-high/HLA-B HED-high (both high) GI patients experienced better OS than other GI cancer patients (Fig. 4e, log-rank test *p* < 0.05).

Collectively, our data confirm the robust prognostic value of the germline divergent allele advantage of the HLA-B gene and its potential joint utility with TMB in predicting efficacy and clinical outcomes in GI cancer.

### Genomic characterization of high HLA-B HED tumors in patients with HLA-B heterozygosity

Higher HED is expected to increase the diversity of HLA-I presented neopeptide repertoires. Moreover, a previous report has described an association between the mean HED and the abundance of neopeptides among patients fully heterozygous at HLA-I genes [18]. We therefore aimed to explore how HLA-B HED correlates with somatic genomic features in GI cancer (Additional file 3: Fig. S8). Interestingly, in the subgroup analysis limited to patients who were heterozygous at the HLA-B locus as described previously [18], we observed no significant association between HLA-B HED and other genomic features, such as CNA burden, TMB, and tumor neoantigen burden (TNB) (Additional file 3: Fig. S8).

**Fig. 5:**
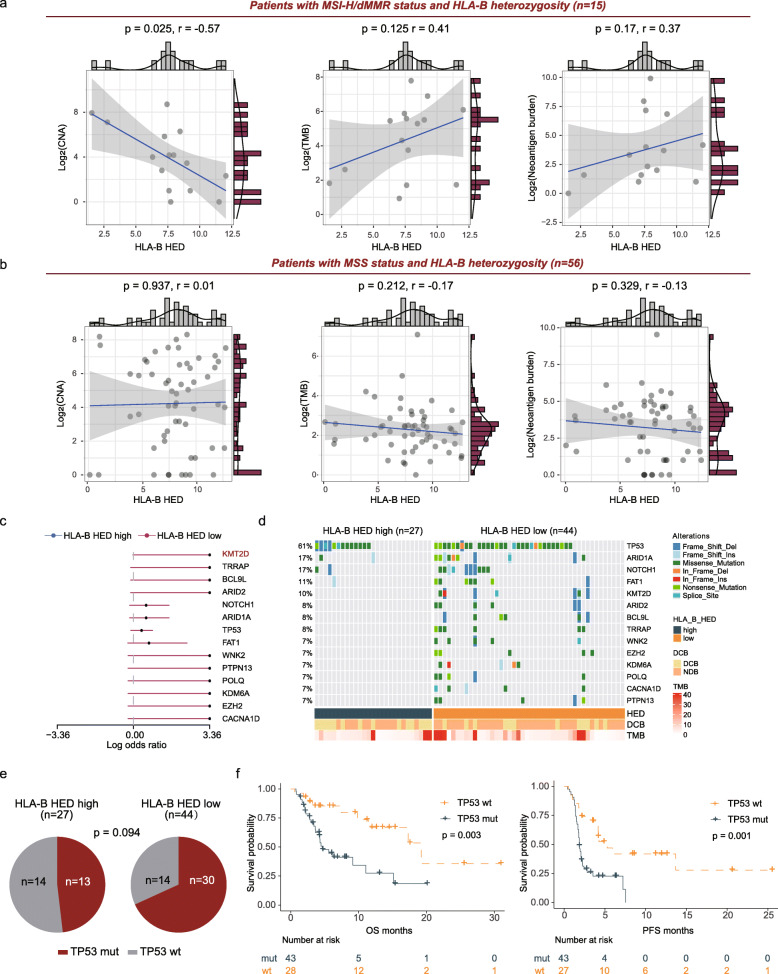
Correlation of HLA-B HED with genomic determinants and mutational patterns in patients with HLA-B heterozygosity. **a**, **b** Correlation of HLA-B HED with the CNA burden, TMB and neoantigen burden in the MSI-H (**a**) or MSS (**b**) subpopulations (two-sided Spearman’s correlation) in patients with HLA-B heterozygosity (*n* = 71). **c** Mutation frequency of driver genes between the high- and low-HLA-B HED subgroups in patients with HLA-B heterozygosity (*n* = 71) were compared using mafCompare function of the maftools R package. **d** Oncoplot of the potentially differentially mutated driver genes. **e** Association of HLA-B HED with TP53 mutations. **f** Kaplan-Meier survival analysis of OS and PFS between patients with or without TP53 mutations in patients with HLA-B heterozygosity (PFS information was not available for one patient). HLA-B HED high was designated as HLA-B HED > 8.61

As previously reported, MSI-H/dMMR tumors represent a distinct phenotype that harbors a large number of somatic mutations and mutation-associated neoantigens [53]. We therefore subclassified our cohort according to the MSI/MMR status of tumor samples among patients heterozygous at the HLA-B gene (*n* = 71). Intriguingly, a trend towards a positive correlation between HLA-B HED and TMB (*r* = 0.41, *p* = 0.125) as well as TNB (*r* = 0.37, *p* = 0.17) was found in the MSI-H/dMMR subset (Fig. 5a). Furthermore, a strong negative correlation was also identified between CNA burden and HLA-B HED (Fig. 5a, *r* = − 0.57, *p* = 0.025). On the contrary, in the MSS subset, no significant correlation was detected among all genomic features (Fig. 5b). These observations suggest that HLA-B HED might be associated with increased genome instability and the diversity of neopeptide repertoire in MSI-H/dMMR GI tumors. However, our limited sample size and power call for larger studies.

It was hypothesized that the oncogenic mutational landscape could be restricted during tumor development in a manner that is dependent on the sub-peptidome presented by each individual’s HLA molecules [54]. As HED was calculated by measuring the Grantham distance between the peptide-binding domains of the two alleles, even among patients who are fully heterozygous at each of the HLA-I genes, the physiochemical sequence divergence between alleles still varies substantially. We thus compared the mutational frequencies of cancer driver genes between the high- and low- HLA-B HED subgroups in patients with HLA-B heterozygosity (*n* = 71). Interestingly, we observed that multiple driver genes, including lysine methyltransferase 2D (KMT2D), TP53, AT-rich interaction domain 1A (ARID1A), and NOTCH1, tended to show a higher prevalence (Fig. 5c–e, *p* < 0.2) in the HLA-B HED low subgroup versus the high subpopulation. Among these driver genes, the prevalence of KMT2D reached a statistical significance (Fig. 5c, *p* < 0.05) and the differential mutational frequency of TP53 showed a definite trend (Fig. 5e, *p* = 0.094). More importantly, TP53 mutation was associated with worse OS and PFS in GI cancer patients after ICB treatment (Fig. 5f, log-rank *p* < 0.01 for all comparisons). Additionally, univariate and multivariate cox regression analysis showed that HLA-B HED, TP53 mutation, and TMB are all independent prognostic factors for these patients (Additional file 3: Fig. S9). Altogether, we showed here that increased HLA-B sequence diversity may correlate with different mutational characteristics.

### Immunophenotypic changes associated with high sequence divergence at the HLA-B locus

**Fig. 6:**
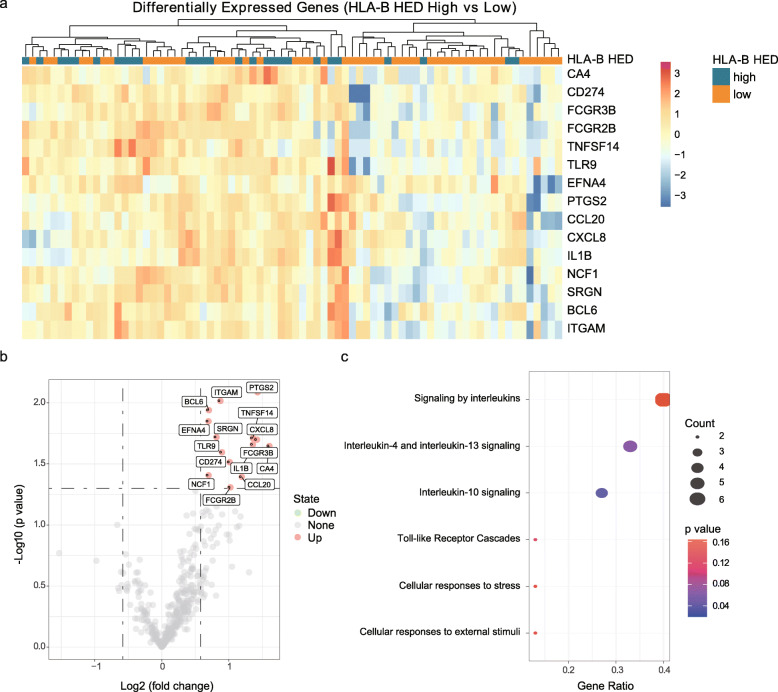
Correlation between HLA-B HED and the expression of immune-related genes. **a** Heatmap of differentially expressed genes (DEGs) between the low HLA-B HED and high HLA-B HED subgroups in the PUCH cohort. DEGs were obtained from a 395-plex RNA immune oncology (RNA IO) profiling platform. **b** Volcano plot of DEGs. **c** Pathway enrichment analysis of DEGs by using the enrichPathway function from the ReactomePA R package

Finally, we applied a RNA IO profiling platform to assess the associations between HLA-B HED and immunophenotypic changes in the tumor microenvironment (Fig. 6). When comparing the 395 immune-related gene profiles between two distinct subgroups as high HLA-B HED (*n* = 27) and low HLA-B HED (*n* = 49), we detected a strong enrichment of immune-related genes (Fig. 6a). Strikingly, no downregulated genes were identified in the high HLA-B HED subgroup versus the low HLA-B HED subgroup (Fig. 6b). Moreover, the upregulated genes in the high HLA-B HED samples mainly fall into the following pathways, including signaling by interleukins, interleukin-4 and interleukin-13 signaling, and interleukin-10 signaling (Fig. 6c). These observations indicate that patients with high HLA-B HED may represent an immune-inflamed phenotype, which might be associated with the improved efficacy of ICB treatment in GI cancer patients.

## Discussion

Heterozygosity or high sequence divergence at the HLA-I locus has been shown to be indicative of favorable clinical outcomes after ICB immunotherapy, particularly in melanoma and NSCLC [17, 18]. In our investigation, we identified that GI cancer patients with a high level of germline sequence divergence at the HLA-B gene experienced improved efficacy and favorable clinical outcomes. Furthermore, the joint utility of germline HLA-B HED and TMB may better stratify GI cancer patients into benefited and non-benefited subgroups.

To explore the clinical relevance of host genetic factors during ICB immunotherapy in GI cancer, we comprehensively analyzed the effects of both zygosity and HED at the HLA-A, HLA-B and HLA-C loci on the clinical outcomes of two independent GI cancer cohorts. Interestingly, heterozygosity in each HLA class I locus revealed no correlation with efficacy or outcome in the PUCH cohort. Most recently, Marcelo et al. also showed no significant correlations for HLA class I zygosity and PFS or OS in three independent cohorts of patients with NSCLC received ICB treatment [55]. These observations are distinct from the large pan-cancer cohort analysis demonstrating the influence of the HLA genotype in ICB-treated patients [17]. One possible explanation for this finding is that HLA class I zygosity may have a marked impact on the clinical outcomes of patients with melanoma, as patients with melanoma account for ~ 35% of those in the pan-cancer cohort [17]. Here, we present new evidence that sequence divergence at the HLA-B locus is a reliable germline genomic determinant for predicting clinical outcomes in GI cancer patients following ICB treatment, as demonstrated in studies of both the PUCH and MSK GI cohorts. Although a previous investigation showed that the mean HED of the three HLA-I loci strongly influences response to ICB immunotherapy [18], we found that neither HLA-A nor HLA-C showed predictive or prognostic significance in our cohort. Indeed, well-established differences between the HLA class I loci have been found, despite their similar roles in pathogen defense [56]. It appears that HLA-B alleles may display greater diversity than the other two, as the HLA-B alleles might be diversifying more rapidly [57]. Accordingly, we observed that the HED was remarkably higher in HLA-B than in HLA-C, but the difference between HLA-B and HLA-A was not significant. HLA genotypes with more divergent alleles were hypothesized to increase immunocompetence and allow for broader antigen-presentation to immune effector cells. Importantly, an in silico analysis revealed a strong correlation between peptides derived from intracellular pathogens and pairwise sequence divergence in the HLA-B and HLA-C alleles [33]. Taken together, these findings may partially explain why HLA-B functions as a dominant germline genomic correlate for clinical outcomes in ICB-treated GI cancer patients. However, the relative contributions of each HLA-I allele in altering tumor immunogenicity have not yet been evaluated.

Moreover, we assessed the predictive and prognostic value of HLA-B HED in different subgroups and showed that patients with high HLA-B HED levels tended to have better clinical outcomes than those with low HED level regardless of TMB and MSI status. Intriguingly, in the MSI-H patients, we observed a positive trend of association between HLA-B HED and TMB or TNB, although the correlation coefficient did not quite achieve the threshold for statistical significance with the limited sample size. Within MSI-H tumors, TMB appears to be an important independent biomarker [58], as high mutation load leads to the generation of neoantigens presented by HLA-I molecules, which attract cytotoxic T lymphocytes to the tumor microenvironment via TCR engagement with HLA [53, 59]. Here, we showed that germline HLA-B sequence diversity may correlate with the abundance mutation load and neopeptides in MSI-H tumors. In addition, we also found a negative correlation between HLA-B HED and CNA burden. It has been proposed that high level of CNA may weaken some aspects of antigen presentation by HLA molecules, a key step for tumor cells to be recognized by the immune system [60]. However, how the germline HLA genotype interferes or interacts with aneuploidy during tumor progression remains much less explored. Notably, we did not identify any associations between HLA-B HED and TNB in the MSS subgroup. Presumably, these observations may, at least partially, be explained by the mutational heterogeneity across different types of GI cancer from our PUCH cohort. Indeed, substantial difference in the landscape of neoantigen among different cancer types has been reported [61].

Avoiding immune destruction is one of the hallmarks of cancer, suggesting that the functional host immune system is an essential determinant to tumor progression. A previous report has demonstrated that the HLA-I genotype-based binding affinity score can predict which mutations are more likely to emerge in their tumor [54]. Therefore, we investigated whether the oncogenic mutational landscape could be influenced by the sequence divergence of HLA-B loci in our cohort. Interestingly, patients with low HLA-B HED tended to exhibit higher TP53 mutational frequencies, and patients with TP53 mutated cancers experienced unfavorable clinical outcomes than patients within the wildtype subgroup. Our finding was in accordance with prior studies reporting that TP53 mutations play a negative role in antitumor immunity in GI cancer [62]. Theoretically, the binding affinity of the HLA-I complex for peptides is a major determinant of antigenicity, and its diversity may ultimately shape the landscape of oncogenic mutations [54]. Here, our results suggest the influence of HLA-B allele divergence in shaping the mutational frequency of KMT2D, TP53, NOTCH1, and several other driver genes, warranting further research to explore the effect of the HLA-B genotype on the restricted immunoediting pattern during tumor progression.

Finally, we also noted a positive correlation between HLA-B HED and enrichment of immune-related gene expression in tumor samples. Thus far, little is known about the associations between host genetic factors and tumor immune infiltration. Our data provide evidence that higher HLA-B sequence diversity may imply an “inflammatory” phenotype in GI cancer patients. However, further investigation will be needed to unravel the underlying biological mechanisms.

## Conclusions

In summary, our data propose an interesting perspective on the association between germline HLA-B sequence diversity and the immunotherapeutic response in GI cancer. Moreover, patients with high HLA-B HED and high TMB tend to experience favorable clinical outcomes, suggesting the potential utility of a combinatorial biomarker for patient stratification. HLA typing via blood sample DNA sequencing may offer valuable information on how host genetic factors impact the efficacy of immunotherapy in GI cancer, especially when tissue samples suffer from limited accessibility, tumor purity, and heterogeneity.

## Supplementary Information

**Additional file 1: Table S1.** Clinic characteristics of PUCH cohort.

**Additional file 2:.** Supplementary Methods

**Additional file 3: Figure S1.** Effect of HLA class I zygosity on clinical outcomes of GI patients receiving ICB treatment. **Figure S2.** Associations between mean HED and OS in the PUCH cohort. **Figure S3.** Univariate cox regression analysis. **Figure S4.** Association of HLA-I HED with clinical outcomes of GI patients receiving ICB treatment. **Figure S5.** HLA-B HED is associated with improved OS in the MSK GI cancer cohort. **Figure S6.** MSI status is associated with favorable prognosis in the PUCH GI cancer cohort. **Figure S7.** TMB is associated with improved survival in the PUCH GI cancer cohort. **Figure S8.** Correlation of HLA-B HED with genomic determinants in patients with HLA-B heterozygosity. **Figure S9.** Univariate and multivariate cox regression analysis on patients with HLA-B heterozygosity.

## Data Availability

The WES-FASTQ files data were deposited at Genome Sequence Archive, https://bigd.big.ac.cn/gsa-human/browse/HRA000898 (bioProject: PRJCA005338; accession: HRA000898) [63]. Because patients, within the context of therapeutic trials, did not consent to release of raw sequence data for confidentiality or privacy purposes, the data will be available via controlled access by reasonable request. The data generated and analyzed during this study are described in the following data record: the HLA genotype, genomic data, and clinical characteristics are available in the figshare repository: 10.6084/m9.figshare.16607891 [64]; 10.6084/m9.figshare.16607894 [65]. The RNA IO data of is available in the following data record: 10.6084/m9.figshare.16607900 [66]. The data involving the analysis of the MSK-GI cohort is available in the figshare repository: 10.6084/m9.figshare.14179295 [67].

## Abbreviations

ICB: Immune checkpoint blockade
PD-1: Programmed cell death protein 1
PD-L1: Programmed cell death ligand 1
GI cancer: Gastrointestinal cancer
TMB: Tumor mutational burden
MSI: Microsatellite instability
MMR: Mismatch repair
HLA-I: Human leukocyte antigen class I
TCR: T cell receptor
NSCLC: Non-small-cell lung cancer
HED: HLA-I evolutionary divergence
PUCH: Peking University Cancer Hospital & Institute
MSK: Memorial Sloan Kettering Cancer Center
WES: Whole-exome sequencing
PBMC: Peripheral blood mononuclear cell
RECIST: Response Evaluation Criteria in Solid Tumors
DCB: Durable clinical benefit
CR: Complete response
PR: Partial response
SD: Stable disease
NDB: No durable benefit
PD: Progressive disease
OS: Overall survival
PFS: Progression-free survival
NGS: Next-generation sequencing
BAM: Binary Alignment Map
HLA-A: HLA class I allele
CNA: Copy number alteration
SNVs: Single nucleotide variants
FFPE: Formalin fixation and paraffin embedding
MSI-H: Microsatellite instability high
ExAC: Exome Aggregation Consortium
gnomAD: Genome Aggregation Database
dMMR: Deficient mismatch repair
MSS: Microsatellite stability
RPM: Reads per million
HK: Housekeeping
DEG: Differentially expressed genes
TNB: Tumor neoantigen burden
