# Supplementary material for: Germline HLA-B evolutionary divergence influences the efficacy of immune checkpoint blockade therapy in gastrointestinal cancer
Source: Genome Med. 2021 Nov 3;13:175. doi: 10.1186/s13073-021-00997-6 (PMC8567649; doi:10.1186/s13073-021-00997-6)
Supplement: Supplementary file 2 — Additional file 2:. Supplementary Methods [file 13073_2021_997_MOESM2_ESM.docx]

**Additional file 2: Supplementary methods**

**1. HLA genotyping**

Adaptors of raw read pairs were trimmed using Trimmomatic (v0.39). Clean reads were then mapped against the human reference genome (build hg19, UCSC) using Burrows-Wheeler Aligner (BWA) [BWA-MEM (v0.7.12)] and sorted using SAMtools (v1.3). MarkDuplicates followed by local indel realignment was performed using GATK version v2.8. Reads in fastq format within the HLA gene region were extracted from the bam file after MarkDuplicates. Next, the fastq file was input into HLA-HD (v 1.2.0.1) for analysis of HLA allele type (minimum_tag_size 50, rate_of_cutting 0.95). This method consists of constructing an extensive dictionary of HLA alleles. The final data includes the type of HLA alleles, focusing on the classical HLA class I molecules [1].

**2. Whole-exome sequencing and data preparation**

The DNA from all tumor tissues and matched with blood samples were profiled with whole-exome sequencing. In brief, we extracted DNA from FFPE tissue and matched blood cell using the blackPREP FFPE DNA Kit (Analytik Jena AG, Jena, Germany) and the Tiangen Whole Blood DNA Kit (Tiangen, Beijing, PRC), following shearing into fragments with a peak at 200 bp by a Covaris M220 Focused-Ultrasonicator (Covaris, Massachusetts, USA). DNA libraries were constructed with a KAPA HTP Library Preparation Kit and captured using a NimbleGen 44M human exome array. The captured libraries were then sequenced on an Illumina NovaSeq platform. Sequencing data were mapped to the reference genome (hg19) using the BWA programs [2]. Variants were called using VarDict and FreeBayes [3]. The functional annotation of the genetic variants was performed with the ANNOVAR assay [4].

Somatic variant identification was performed according to following filters: (1) the mutations of FFPE tumor samples were blanked by paired blood cell samples from patients. (2) located in intergenic regions or intronic regions; (ii) synonymous SNVs; (iii) depth < 40; (iv) allele frequency < 0.03; and (v) allele frequency ≥ 0.002 in the Exome Aggregation Consortium (ExAC) database. The TMB values (the number of SNVs per Mb) were obtained using a validated algorithm [5].

Based on the somatic SNVs and HLA typing results of its paired germline sample generated via OptiType [6], neoantigens were predicted through software netMHCpan-4.0. To ensure the accuracy, the neoantigens with predicted binding affinity of mutation (Aff_mut) ≤ 500 and Aff_mut/Aff_wild < 1 were selected.

For copy number analysis, blood cell samples from patients were used as paired controls, and the CONTRA assay was used to call copy number variations from the formalin-fixed paraffin-embedded (FFPE) tumor samples for each patient [7]. CNA burden was defined as the total number of genes with copy number gains or losses [8].

**3. RNA Immune Oncology Panel Sequencing and Data Normalization**

We performed RNA immune oncology profiling of each sample as previously described [9]. This panel contains 395 immune-related genes, which could be divided as the following categories: immunological function and response, tumor markers, markers of tumor infiltrating cells, tumor-specific antigens, essential signaling pathways, and housekeeping (HK) genes. RNA was extracted from the FFPE samples, and then reverse transcribed into cDNA, amplified, and ligated to fluorescent barcodes. Libraries were pooled in equal molar amounts and sequenced on the Ion S5 530 chip (Thermo Fisher Scientific). Then 1-2 M reads per sample were obtained for data analysis. Ten housekeeping (HK) genes were applied as endogenous controls. The absolute readout of each HK gene was compared against a predetermined HK reads per million (RPM) profile.

The baseline HK RPM profile was established by averaging the RPM of the GM12878 cell line sample replicates across different sequencing runs. We calculated the fold-change ratio for each HK gene (ratio of HK = absolute read count of HK/RPM profile of HK) and used the average value of all HK ratios as the normalization ratio for the pending sample (normalization ratio = median of all HK ratios). We then calculated the normalized RPM (nRPM) of all genes of each sample with the following formula: nRPM of sample S, gene G = absolute read count of sample S, gene G/normalization ratio of sample S.

**References**

1. Kawaguchi S, Higasa K, Shimizu M, Yamada R, Matsuda F. HLA-HD: An accurate HLA typing algorithm for next-generation sequencing data. Hum Mutat 2017;38(7):788-97.

2. Li H. Aligning sequence reads, clone sequences and assembly contigs with BWA-MEM. arXiv 2013;1303.3997.

3. Lai Z, Markovets A, Ahdesmaki M, Chapman B, Hofmann O, McEwen R, et al. VarDict: a novel and versatile variant caller for next-generation sequencing in cancer research. Nucleic Acids Res 2016;44(11):e108.

4. Wang K, Li M, Hakonarson H. ANNOVAR: functional annotation of genetic variants from high-throughput sequencing data. Nucleic Acids Res 2010;38(16):e164.

5. Chalmers ZR, Connelly CF, Fabrizio D, Gay L, Ali SM, Ennis R, et al. Analysis of 100,000 human cancer genomes reveals the landscape of tumor mutational burden. Genome Med 2017;9(1):34.

6. Szolek A, Schubert B, Mohr C, Sturm M, Feldhahn M, Kohlbacher O. OptiType: precision HLA typing from next-generation sequencing data. Bioinformatics 2014;30(23):3310-6.

7. Li J, Lupat R, Amarasinghe KC, Thompson ER, Doyle MA, Ryland GL, et al. CONTRA: copy number analysis for targeted resequencing. Bioinformatics 2012;28(10):1307-13.

8. Budczies J, Seidel A, Christopoulos P, Endris V, Kloor M, Gyorffy B, et al. Integrated analysis of the immunological and genetic status in and across cancer types: impact of mutational signatures beyond tumor mutational burden. Oncoimmunology 2018;7(12):e1526613.

9. Paluch BE, Glenn ST, Conroy JM, Papanicolau-Sengos A, Bshara W, Omilian AR, et al. Robust detection of immune transcripts in FFPE samples using targeted RNA sequencing. Oncotarget 2017;8(2):3197-205.
