# Supplementary material for: Germline HLA-B evolutionary divergence influences the efficacy of immune checkpoint blockade therapy in gastrointestinal cancer
Source: Genome Med. 2021 Nov 3;13:175. doi: 10.1186/s13073-021-00997-6 (PMC8567649; doi:10.1186/s13073-021-00997-6)
Supplement: Supplementary file 3 — Additional file 3: Figure S1. Effect of HLA class I zygosity on clinical outcomes of GI patients receiving ICB treatment. Figure S2. Associations between mean HED and OS in the PUCH cohort. Figure S3. Univariate cox regression analysis. Figure S4. Association of HLA-I HED with clinical outcomes of GI patients receiving ICB treatment. Figure S5. HLA-B HED is associated with improved OS in the MSK GI cancer cohort. Figure S6. MSI status is associated with favorable prognosis in the PUCH GI cancer cohort. Figure S7. TMB is associated with improved survival in the PUCH GI cancer cohort. Figure S8. Correlation of HLA-B HED with genomic determinants in patients with HLA-B heterozygosity. Figure S9. Univariate and multivariate cox regression analysis on patients with HLA-B heterozygosity. [file 13073_2021_997_MOESM3_ESM.pdf]

Additional file 3: Supplementary figures

Fig. S1. Effect of HLA class I zygosity on clinical outcomes of GI patients receiving ICB treatment.

Fig. S2. Associations between mean HED and OS in the PUCH cohort.

Fig. S3. Univariate cox regression analysis.

Fig. S4. Association of HLA-I HED with clinical outcomes of GI patients receiving ICB treatment.

Fig. S5. HLA-B HED is associated with improved OS in the MSK GI cancer cohort.

Fig. S6. MSI status is associated with favorable prognosis in the PUCH GI cancer cohort.

Fig. S7. TMB is associated with improved survival in the PUCH GI cancer cohort.

Fig. S8. Correlation of HLA-B HED with genomic determinants in patients with HLA-B heterozygosity.

Fig. S9. Univariate and multivariate cox regression analysis on patients with HLA-B heterozygosity.

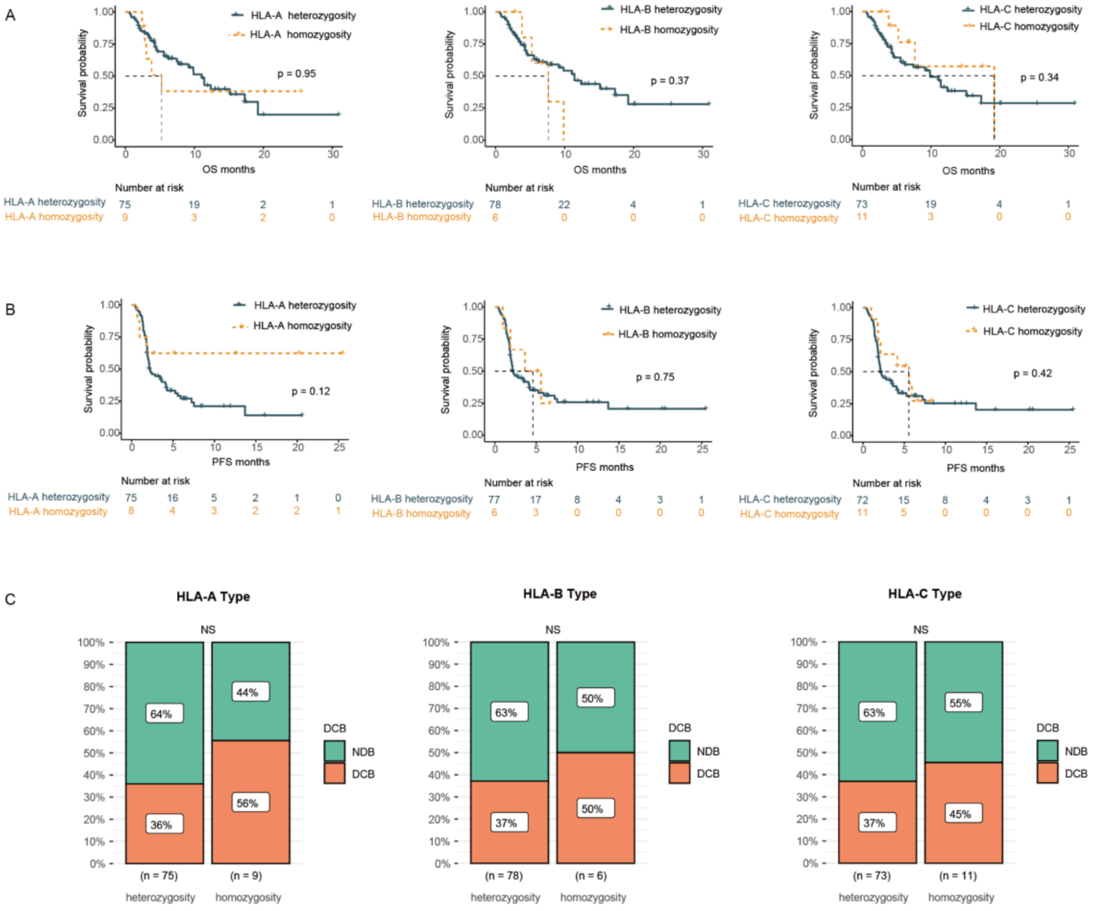

Fig. S1. Effect of HLA class I zygosity on clinical outcomes of GI patients receiving ICB treatment. A-B. Kaplan-Meier survival analysis comparing the OS (**A**) or PFS (**B**) curves between HLA heterozygous and homozygous at each of HLA-A, HLA-B and HLA-C genes. **C.** Proportions of patients with DCB within heterozygous- and homozygous- at each of the HLA-A, HLA-B and HLA-C genes (ns: $p > 0.05$, chi-squared test).

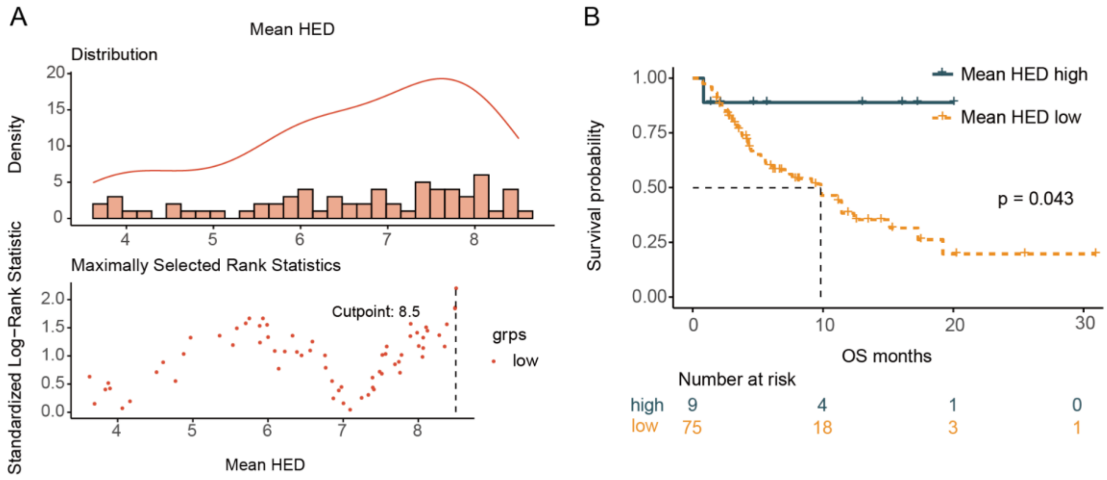

Fig. S2. Associations between mean HED and OS in the PUCH cohort. A. Optimal cutpoint of mean HED for OS survival analysis was analyzed by using the "surv_cutpoint" function of the "survminer" R package. **B.** Kaplan-Meier survival analysis comparing OS between patients with high and low mean HED. The mean HED was calculated as the mean of the three pairwise divergences of HLA-A, HLA-B and HLA-C.

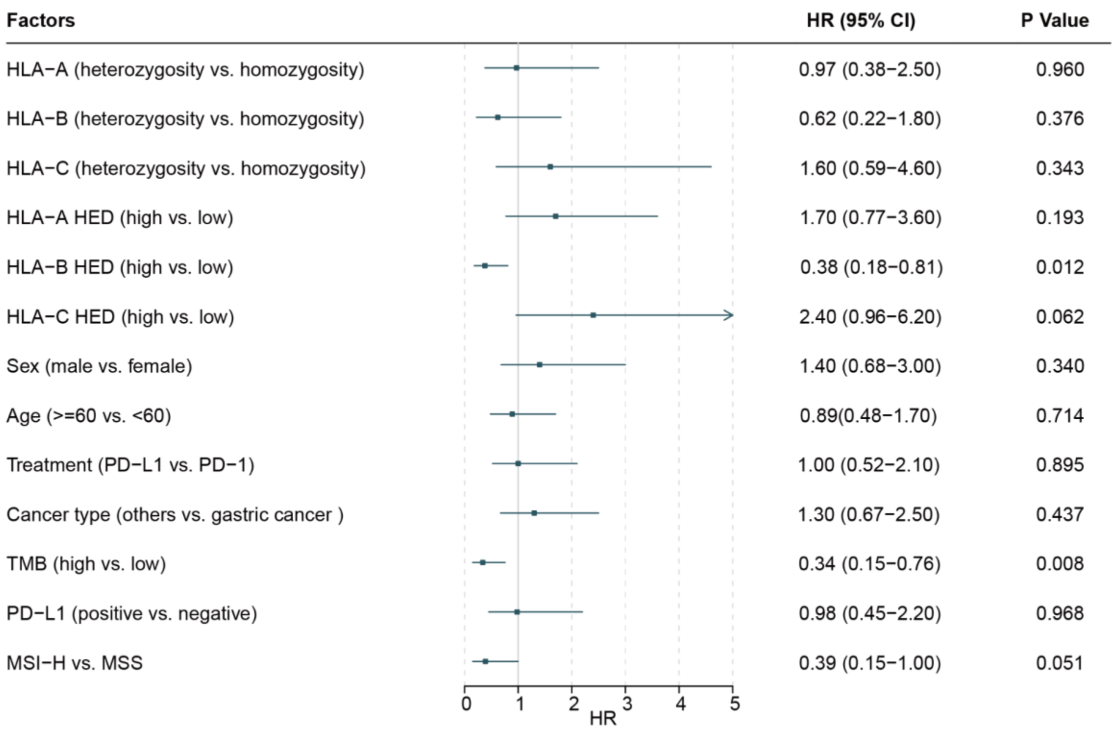

Fig. S3. Univariate cox regression analysis. For the PUCH cohort, we performed a univariate cox regression analysis on various factors including clinical, germline HLA, TMB, MSI and PD-L1 (TMB and MSI information was available for 76 patients). The optimal cutpoints for each HED were determined by using the maximally selected rank statistics (‘maxstat’ method from the “surv_cutpoint” function of the “survminer” R version 3.6.1). The optimal cutoff point for TMB was defined by using the Youden index in receiver operating characteristic (ROC) analysis for DCB.

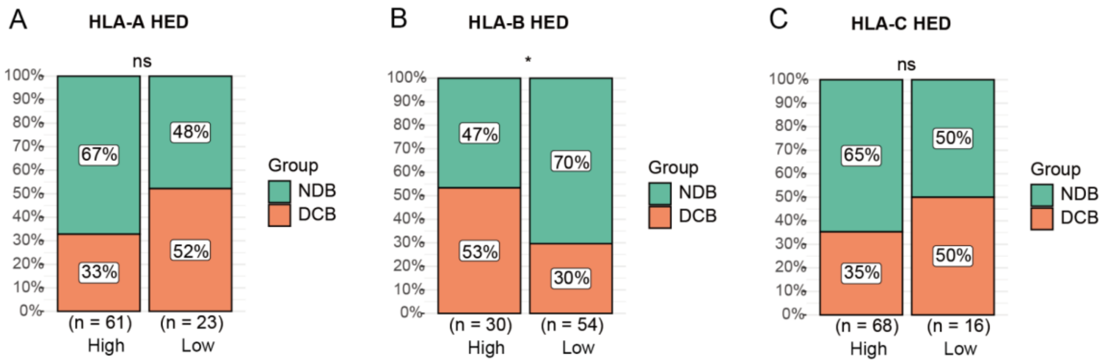

Fig. S4. Association of HLA-I HED with clinical outcomes of GI patients receiving ICB treatment. A-C. Proportions of patients with DCB within the high- and low- HED subgroups at each of the HLA-A (**A**), HLA-B (**B**) and HLA-C (**C**) loci (chi-squared test). Patients were dichotomized into low-HED and high-HED subgroups with differential risks for OS using the optimal cutoff values determined by the "surv_cutpoint" function of the "survminer" R package. *p <0.05.

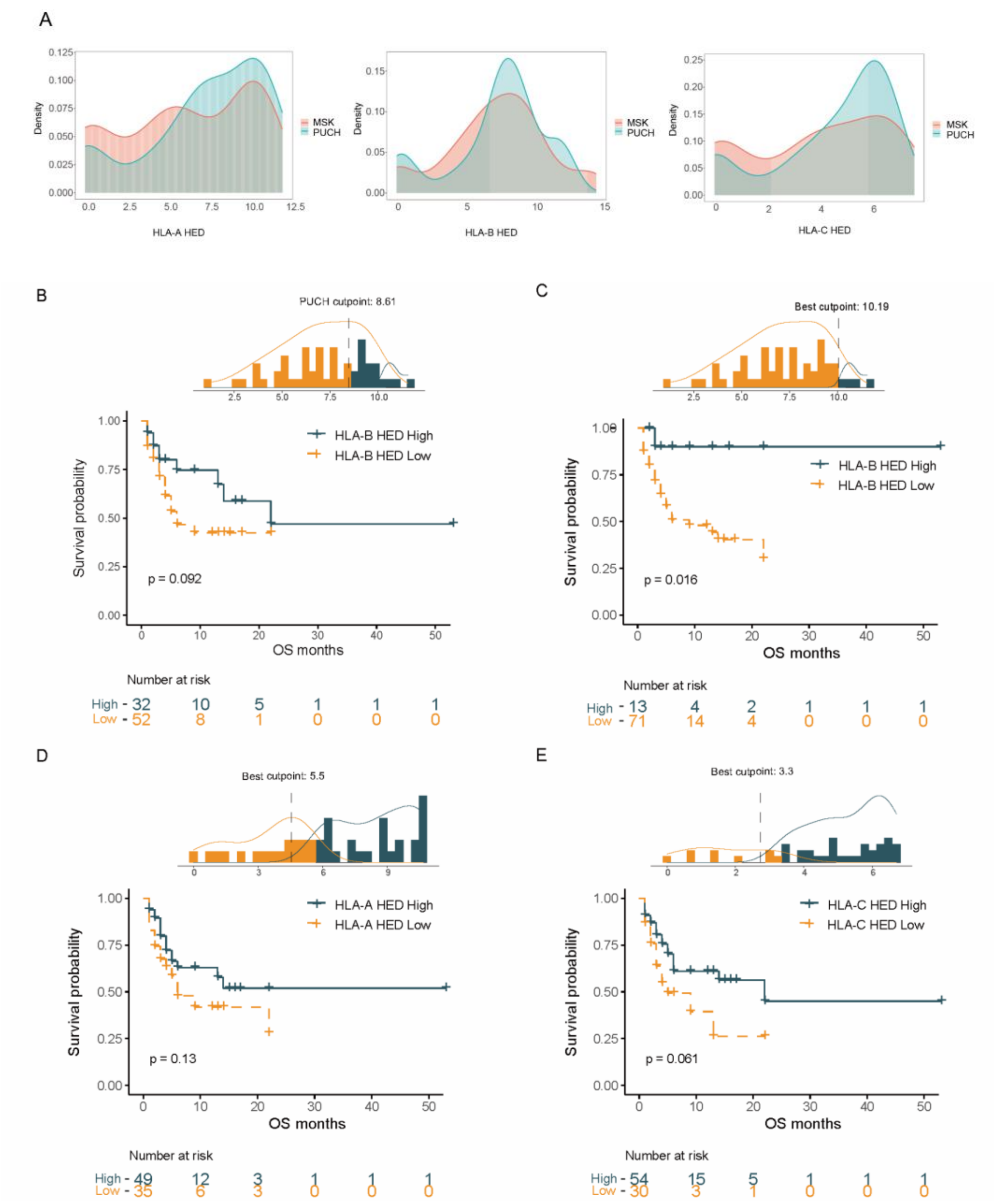

Fig. S5. HLA-B HED is associated with improved OS in the MSK GI cancer cohort. A. Distribution of patient HED across all GI cohorts treated with ICBs (PUCH and MSK GI cohorts). **B.** Association of high HLA-B HED with OS in the MSK GI cancer cohort of patients with ICB treatment. All patients were dichotomized into low- and high- HED subgroups using the same cutoff value as the PUCH cutpoint of 8.61. **C-E.** Kaplan-Meier survival analysis comparing OS between patients with high and low HED at each locus: HLA-B (**C**), HLA-A (**D**) and HLA-C (**E**). Patients from the MSK cohort were dichotomized into low-HED and high-HED subgroups by using the optimal cutoff values determined by the "surv_cutpoint" function of the "survminer" R package. For all comparisons, a two-sided log-rank test was performed.

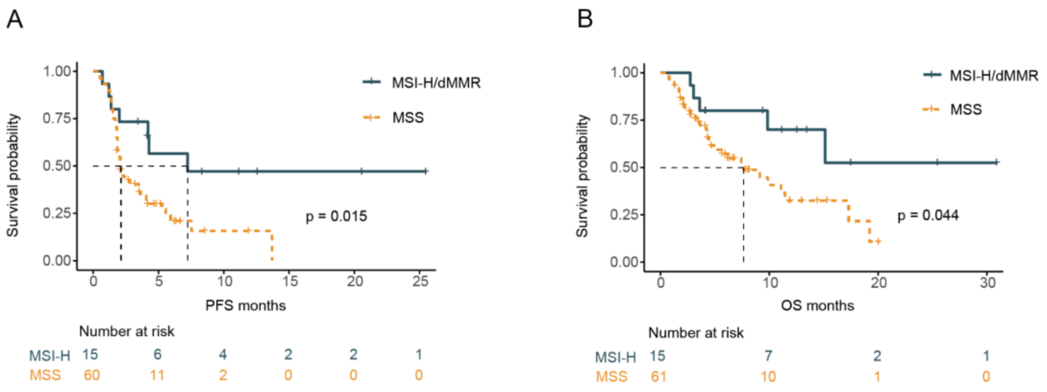

Fig. S6. MSI status is associated with favorable prognosis in the PUCH GI cancer cohort. A, B. Association of MSI status with PFS (**A**) and OS (**B**) in the PUCH GI cancer cohort of patients with ICB treatment.

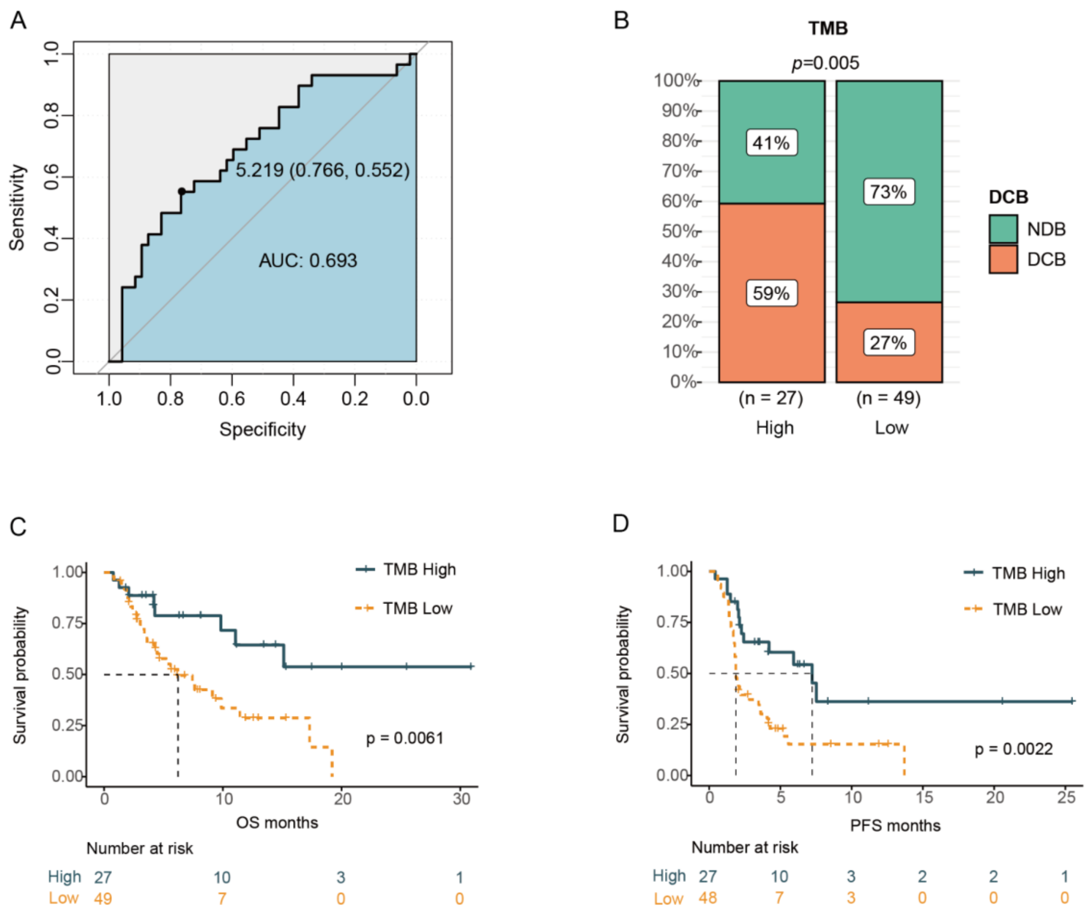

Fig. S7. TMB is associated with improved survival in the PUCH GI cancer cohort. **A.** Receiver operating characteristic (ROC) curve measuring the predictive value of the TMB. The optimal cutoff point for TMB (5.22 mut/Mb) was defined by using the Youden index in ROC analysis for DCB. **B.** Proportions of patients with DCB within the high- and low- TMB subgroups. **C-D.** Association of TMB-high with OS (**C**) and PFS (**D**) in the PUCH GI cancer cohort of patients with ICB treatment. TMB high was designated as TMB > 5.22 mut/Mb.

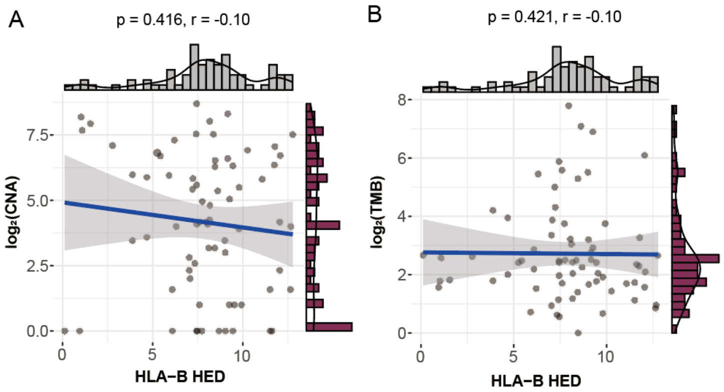

Fig. S8. Correlation of HLA-B HED with genomic determinants in patients with HLA-B heterozygosity. **A-C**. Correlation of HLA-B HED with the CNA level (**A**), TMB (**B**) and neoantigen burden (**C**) in the PUCH GI patients (two-sided Spearman's correlation).

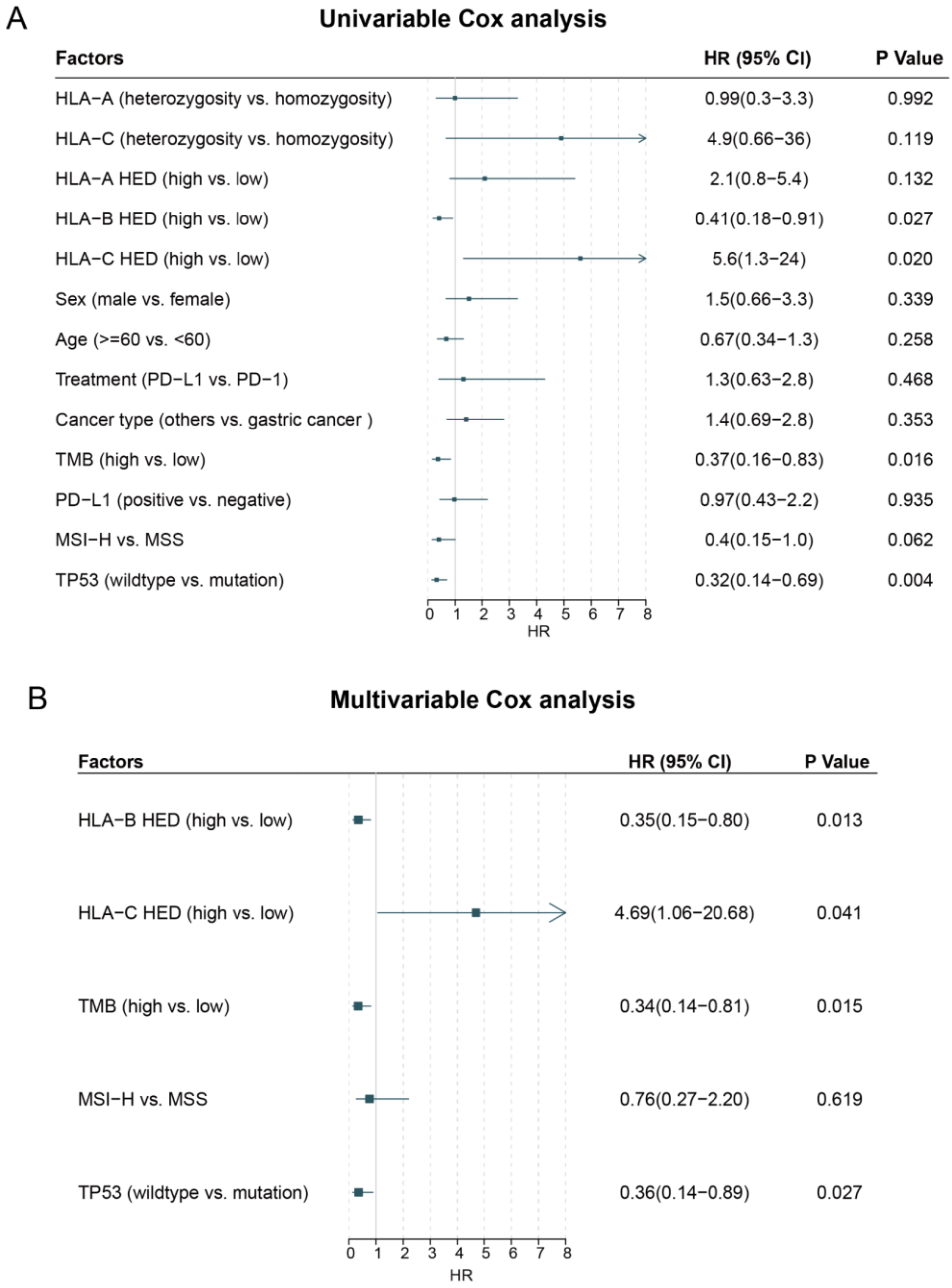

Fig. S9. Univariate and multivariate cox regression analysis on patients with HLA-B heterozygosity. **A**. For patients with HLA-B heterozygosity in the PUCH cohort (n=71), we performed a univariate cox regression analysis on various factors including clinical, germline HLA, TMB, MSI and PD-L1. **B.** Forest plot showing the HRs and 95% CIs for the associations of potential prognostic factors (HLA-B HED, HLA-C HED, TMB, TP53 mutation status and MSI) with OS in multivariable Cox proportional hazards model in all 71 patients with HLA-B heterozygosity. HLA-B HED high, HLA-B HED >8.61; TMB high, TMB >5.22 mut/Mb.
